# Purification of complex samples: Implementation of a modular and reconfigurable droplet-based microfluidic platform with cascaded deterministic lateral displacement separation modules

**DOI:** 10.1371/journal.pone.0197629

**Published:** 2018-05-16

**Authors:** Eloise Pariset, Catherine Pudda, François Boizot, Nicolas Verplanck, Frédéric Revol-Cavalier, Jean Berthier, Aurélie Thuaire, Vincent Agache

**Affiliations:** Univ. Grenoble Alpes, CEA, LETI, DTBS, Grenoble, France; Texas A&M University College Station, UNITED STATES

## Abstract

Particle separation in microfluidic devices is a common problematic for sample preparation in biology. Deterministic lateral displacement (DLD) is efficiently implemented as a size-based fractionation technique to separate two populations of particles around a specific size. However, real biological samples contain components of many different sizes and a single DLD separation step is not sufficient to purify these complex samples. When connecting several DLD modules in series, pressure balancing at the DLD outlets of each step becomes critical to ensure an optimal separation efficiency. A generic microfluidic platform is presented in this paper to optimize pressure balancing, when DLD separation is connected either to another DLD module or to a different microfluidic function. This is made possible by generating droplets at T-junctions connected to the DLD outlets. Droplets act as pressure controllers, which perform at the same time the encapsulation of DLD sorted particles and the balance of output pressures. The optimized pressures to apply on DLD modules and on T-junctions are determined by a general model that ensures the equilibrium of the entire platform. The proposed separation platform is completely modular and reconfigurable since the same predictive model applies to any cascaded DLD modules of the droplet-based cartridge.

## Introduction

Deterministic lateral displacement (DLD) is a microfluidic sample preparation technique that enables size-based separation of particles. As a passive and label-free purification technique, DLD has a high potential to address both micrometer- and nanometer-sized biological particles. Examples of applications are the isolation of circulating tumor cells (CTCs) from blood samples [1][2][3][4][5], the extraction of white blood cells (WBCs) [6][7][8][9][10][11], the manipulation of red blood cells (RBCs) [12][13][14][15][16][17], the isolation of cells [18][19], platelets [20], fungal spores [21], bacteria [22], parasites [23], and the fractionation of isolated extracellular vesicles into several subpopulations [24][25].

DLD separation is induced by an array of regularly disposed pillars to orient the particle-containing fluid path in a microfluidic channel. While flowing in the DLD channel, the trajectory of the particles is determined by their position relatively to the streamline distribution around the pillars. Particles larger than a cutoff diameter—also known as critical diameter (D_c_)—are deviated along the pillar rows (displacement trajectory), while smaller particles remain in the channel axis (zigzag trajectory). At the DLD device outlet, two populations of particles can be isolated around the critical diameter D_c_. The value of D_c_ is determined by the geometrical characteristics of the pillar array, such as the inter-pillar spacing, the slant angle, the channel width and the pillar shape and orientation.[26][27][28] Therefore, the DLD design can be customized to obtain the desired critical diameter according to the final application.

However, most biological samples contain a wide range of particle sizes (from the nanometer scale to the micrometer scale). Therefore, clogging issues make it inadequate to use a unique DLD system to remove all the large contaminants of the sample. For example, isolating exosomes from whole blood requires the successive removal of particles from several tens of micrometers down to a few hundreds of nanometers.

In order to progressively purify complex biological samples through DLD, the most common strategy is to cascade different separation modules into a single chip [1][15][20][23][29][30][31]. However, this strategy presents some limitations due to the fabrication process. Because of the small pillar dimensions that are required to address biological particles with DLD, the fabrication process flow is based on silicon microtechnologies. Due to limitations in terms of diversity of achievable aspect ratio and interpillar spacing implemented across a same wafer, a single chip cannot contain all the pillar dimensions that are required to sort biological particles from the nanometer range up to the micrometer range. Another limitation of this approach is related to the system integration. Indeed, when a single multi-stage DLD chip is developed, this does not enable to target different applications with the same device. In this paper, a modular and reconfigurable platform is proposed to cascade different DLD chips, according to the application of interest. Therefore, any DLD modules can be connected together through the same microfluidic cartridge to separate all the particle groups of the initial sample.

To our knowledge, the first existing strategy to date to connect DLD steps in an integrated platform was presented by Karabacak et al. [2]. They proposed a CTC-iChip with two cascaded devices to isolate CTCs from whole blood: a first DLD step is connected to a second device downstream that contains both inertial focusing and magnetophoresis. The main problematic when connecting a DLD module to another microfluidic stage is related to flow velocity balancing considerations. Indeed, DLD separation is based on the streamline distribution around the pillars. It is thus critical to ensure hydraulic resistance balance at the DLD outlets. If different resistances apply on the DLD outlets, all the streamlines and all the particles will follow the path presenting the lowest resistance. Connecting a following step to a DLD module involves hydraulic resistance imbalance at the DLD outlets: an additional resistance is applied on the outlet connected to the second module (and containing the particles of interest) while the other outlets called “waste” are not connected to the same module. In order to compensate for the output flow rate imbalance, Karabacak et al. [2] connected a syringe pump to the waste channel of a two-outlet DLD device. However, this solution cannot be implemented when all the particles from the different output channels have to be collected in flow for further analysis on chip. Indeed, in the CTC-iChip [2], particles flowing through the waste channel are lost in an external waste syringe. In comparison, the advantage of our platform is the ability to collect particles from all DLD outlets in flow. Therefore, our system can integrate any DLD modules and analyze all the sorted particles in flow on the same cartridge, without external sample manipulation.

A droplet-based solution with T-junctions intersecting the DLD outlets is presented to balance output DLD backpressures in a completely modular and reconfigurable sample preparation device. Our platform is composed of silicon-based DLD chips connected to a plastic-based cartridge with T-junction channels supplied by an additional oil solution to generate water-in-oil droplets. Three configurations using this droplet-based technology are proposed, with a particular example of two cascaded DLD modules. In this configuration, a general predictive model has been developed in order to anticipate the optimized input pressure parameters maximizing the separation efficiency at each step.

## Materials and methods

### Fabrication of the silicon-based devices

The chips are fabricated on 200 mm silicon wafers and they are cut into 22 x 22 mm individual square pieces, each containing 4 DLD devices. The fluidic channels and the inlet/outlet ports are defined by contact photolithography and Reactive Ion Etching (RIE) on a 3μm-thick silicon dioxide (SiO_2_) hard mask (2μm thermal SiO_2_ followed by a deposition of 1μm SiO_2_). Following hard mask etching, fluidic ports are partially etched using deep UV photolithography and RIE. After stripping off the resist, the microchannels are etched through the hard mask using RIE and the holes are etched until the bottom 2μm-thick thermal SiO_2_ layer at the same time. After removing the oxide and cleaning the substrate, a second thermal oxide layer (100 nm thick) is grown and a top 500 μm-thick borosilicate glass cover is sealed by anodic bonding.

### Fabrication of the microfluidic cartridge

The chips were packaged on a custom COC (cyclic olefin copolymer) Fluidic Circuit Board (FCB) with plug and play tubing connectors. Fluidic sealing between the silicon chip and the plastic cartridge was achieved by a magnetic frame holding silicone-based gaskets. The cartridge channels were micromachined and then thermally sealed with a COC cover. T-junction geometries were part of the cartridge channels, with 500 μm x 500 μm cross-sections. In order to soften COC roughnesses on the walls of the microfluidic channels after milling, a solution of 1/3 toluene—2/3 acetone was injected in the channels before rinsing with acetone, ethanol and water. In addition to the aqueous solution coming from the DLD chip, a corn oil solution (Sigma-Aldrich, C8267) was injected in the cartridge at the T-junctions to generate water-in-oil droplets. The association of the silicon DLD chip and the plastic cartridge containing T-junction areas is called the platform.

### Experimental test bench

Imaging was performed by epifluorescence microscopy (Olympus, BX60) with a built-in 100 W mercury lamp (Osram, HBO 103W/2). A standard FITC (fluorescein isothiocyanate) filter cube (Olympus, U-MSWB2) was used to detect fluorescent beads. Imaging was performed with a monochrome fluorescence CCD camera (Olympus, XM10). Sequences of images were superimposed and analyzed with ImageJ software to visualize the trajectory of particles flowing in the DLD channel. Fluids were actuated by a pressure-based flow controller (Fluigent, MFCS-EZ) with input pressures from 50 mbar to 1 bar according to the DLD design in order to obtain a flow rate of about 100 μL/min in the pillar array. The flow rate was chosen to obtain a Stokes flow and avoid flow stream mixing. In the Stokes regime, the flow rate does not influence the DLD separation of particles.

### Protocol

10 μm and 5 μm-monodisperse fluorescent polystyrene beads (ThermoFisher Scientific, Fluoro-Max Dyed Green Aqueous Fluorescent Particles) were used to characterize the DLD devices. As recommended by the manufacturer, the beads were suspended in a 1% surfactant solution (Tween 20, Sigma-Aldrich) in filtered DPBS 1x (gibco life technologies, 14190144) in order to prevent particle aggregation. The solution injected in the “buffer” inlet of DLD modules was also a 1% surfactant solution in filtered DPBS 1x. The oil solution injected at the T-junction was corn oil (Sigma-Aldrich, C8267).

## Results and discussion

### Droplet T-junctions as pressure controllers at DLD outlets

**Fig 1:**
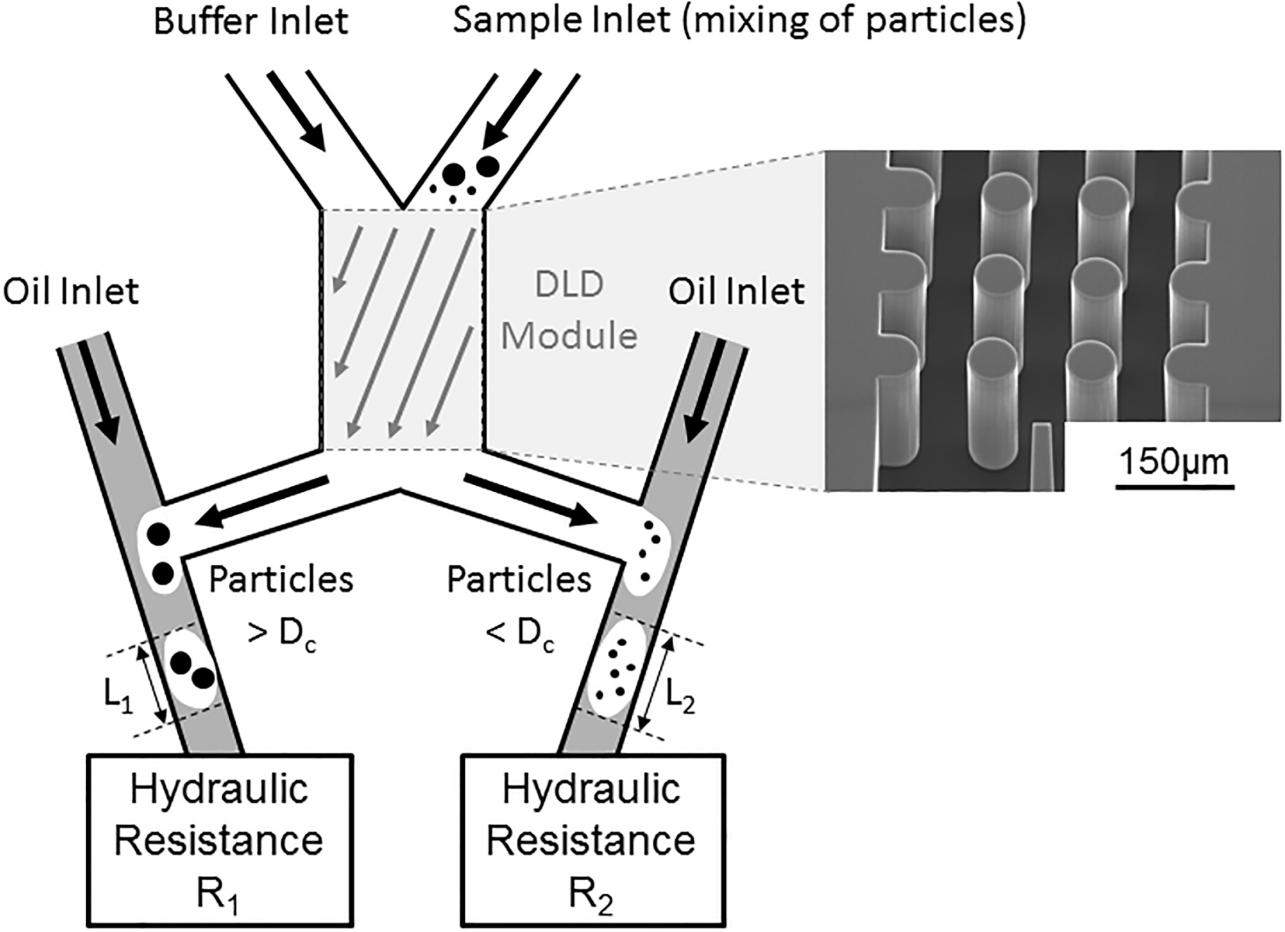
Schematic of the droplet-based pressure control principle. A mixing of particles of different sizes is injected in the right inlet of a DLD module. Particles larger than the critical diameter (D_c_) are deviated towards the left outlet, while smaller particles flow in the right outlet. Each DLD outlet is connected to a T-junction with an oil inlet, for generation of droplets of lengths L_1_ and L_2_. Additional microfluidic modules are connected to the T-junction, with hydraulic resistances called respectively R_1_ and R_2_.

In the presented platform, droplet formation at T-junctions is proposed as a new solution to control hydraulic pressures. Indeed, generating droplets at a T-junction enables to apply a specific pressure on the dispersed phase thanks to an additional continuous phase. In our DLD problematic, two output hydraulic resistances have to be controlled and balanced at both DLD outlets (Fig 1): the left outlet that contains large deviated particles, and the right outlet that contains smaller, non-deviated particles. These two outlets are generally connected to different downstream microfluidic stages. For example, the left output channel could be connected to an integrated sensor in order to analyze the deviated particles, while the right output channel could be connected to another DLD module for further purification of the sample with a smaller critical diameter. Therefore, the two outlets are usually connected to microfluidic steps with different hydraulic resistances, called R_1_ and R_2_. In this case, a solution is required to balance both output pressures in order to optimize the sorting efficiency of the DLD stage. Our droplet-based approach can also be applied to DLD modules with more than two outlets, by connecting a T-junction to each of the DLD output channel.

The proposed solution is the use of droplets as pressure controllers. Thanks to an additional immiscible phase—called “oil inlet” in Fig 1—the aqueous phase leaving from DLD outlets is encapsulated in T-junctions. By changing the input oil pressure at the T-junction, the end pressure applied on the DLD aqueous phase is locally controlled [32]. This solution has several advantages when compared to the direct pressure control at the DLD outlets (such as connecting a syringe pump to the outlet [2]). First, it enables to keep all the sorted particles in flow inside the cartridge. This is particularly useful when downstream DLD or analytical sensor modules are also integrated in the same microfluidic system. Moreover, encapsulating particles can be very advantageous for various down-stream particle analysis techniques [33][34][35][36][37][38]. Finally, the oil sections between each analyzed droplet can also be exploited as calibration references, for example when the analysis module measures particle density [39]. Indeed, the baseline of the density sensor can be regularly calibrated in this case, thanks to the oil phase of known density.

In case the injection of an oily continuous phase in the downstream analysis module is problematic, it can be considered to use an aqueous solution instead of the oil solution to form water-in-water droplets [40]. Therefore, pressure control through droplet generation can be adaptable to different applications and analysis techniques.

Yan et al. [41] have numerically shown that the pressure at the junction point—at the interface between the dispersed and the continuous phases—fluctuates periodically during the whole generation cycle. For given input pressures, capillary number, contact angle and channel geometry, the local pressure at the junction point fluctuates at the same rate as the droplet formation process. It was shown that the evolution of the junction point pressure is dependent on the droplet generation regime [41]. In particular, the amplitude of the pressure fluctuations during the cycle decreases in the order: dripping regime, squeezing regime, jetting regime and parallel flow regime. In our case, it is important to minimize pressure fluctuations on the DLD aqueous phase, in order to simplify pressure balancing at both sides. On the other hand, jetting and parallel flow regimes do not allow to apply sufficiently high pressures to balance the important hydraulic resistance of DLD modules. From these two considerations, a squeezing regime was chosen for our droplet-based pressure control platform. In this regime, the break-up process is determined by the pressure drop across the droplets, which form in a plug-like shape [42].

In the next sections, it will be demonstrated that the droplet generation at T-junctions can be modeled as an additional hydraulic resistance that is applied on the DLD output solution. The droplet-associated resistance is considered as a constant average value. This approximation is valid because the period of the droplet generation cycle—in the order of 10 milliseconds in the squeezing regime [41]—is significantly lower than the processing time of DLD—in the order of several minutes at a flow rate of 100 μL/min.

### Possible droplet platforms

Two platforms were first implemented with one single DLD module presenting two output channels. This DLD module has 20 μm triangular pillars (D_p_), 20 μm inter-pillar spacing (G) and an array periodicity (N) of 50, with a corresponding critical diameter (D_c_) of 3.5 μm. Each platform makes use of the droplet-based pressure control system with T-junction geometries in the cartridge. In both platforms, two T-junctions are used so that each output DLD channel can be connected to an oil inlet. These platforms enable to balance both backpressures when the DLD outlets are connected to two different microfluidic modules (for example two different analysis systems, adapted to each particle size coming from the DLD outlets).

**Fig 2:**
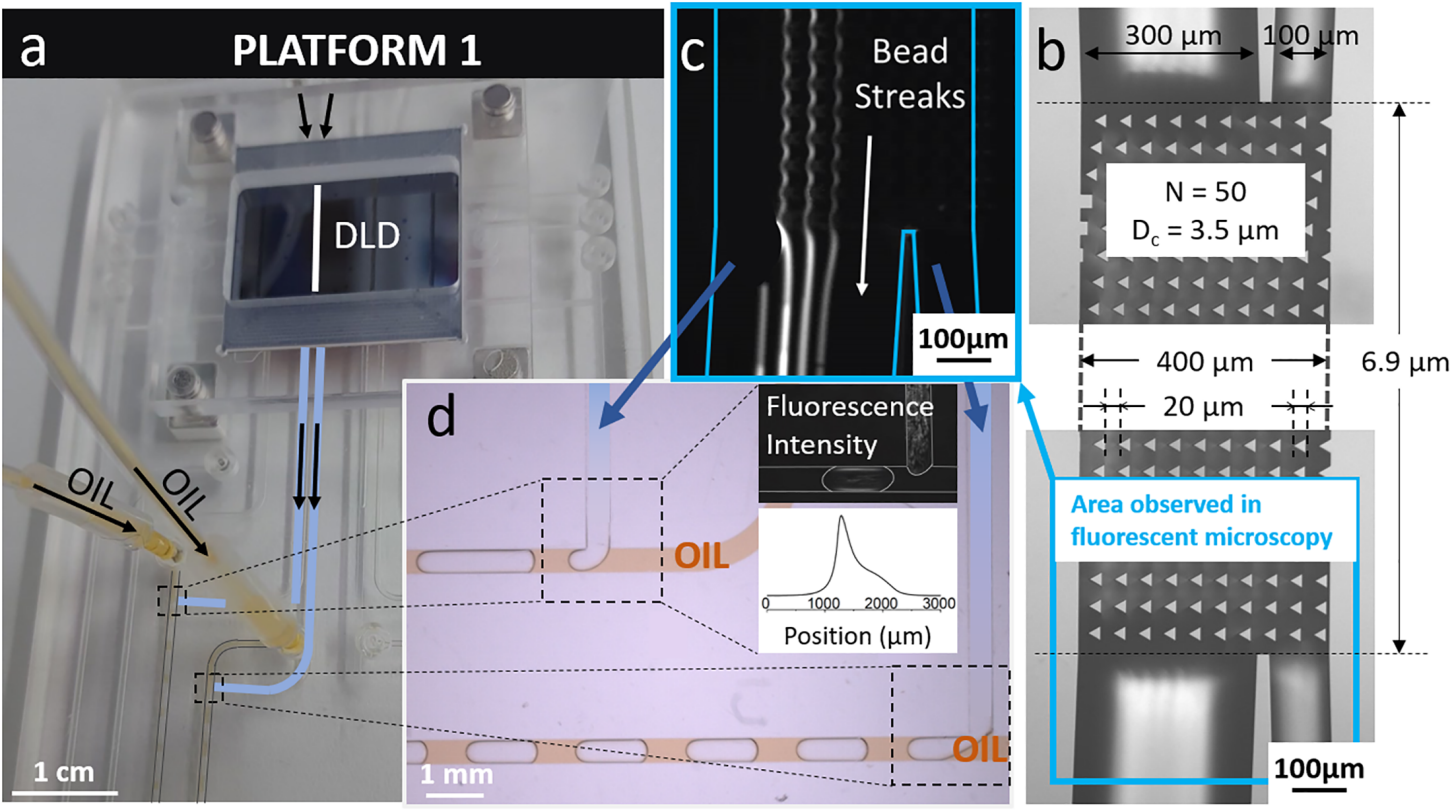
a) Photograph of platform 1 showing the DLD silicon chip connected to a plastic cartridge with two independent T-junctions supplied by two oil inlets. b) Bright-field image of the DLD device with characteristic dimensions. The area located in the blue frame corresponds to the fluorescence image in c). c) Fluorescent streaks of 10 μm-beads at the DLD outlet. d) T-junctions connected to the DLD outlets and fluorescent profile of the channel containing the droplets.

In platform 1 (Fig 2), T-junctions are independent, which means that two oil inlets are required to supply each droplet generation area. When oil pressures are not applied, the system has an intrinsic resistance imbalance at the outlets since the DLD output channels do not have the same width. Therefore, application of oil pressures is required to balance output resistances and direct the bead streaks towards the correct outlet (Fig 2c). The equilibrium is reached for the generation of droplets of different sizes at both DLD outlets (Fig 2d). It was verified that the fluorescence intensity of the beads was focused inside the droplets at the T-junctions. Indeed, no fluorescence was detected in the oil solution (Fig 2d), which suggests no bead escape from the water phase to the oil phase.

**Fig 3:**
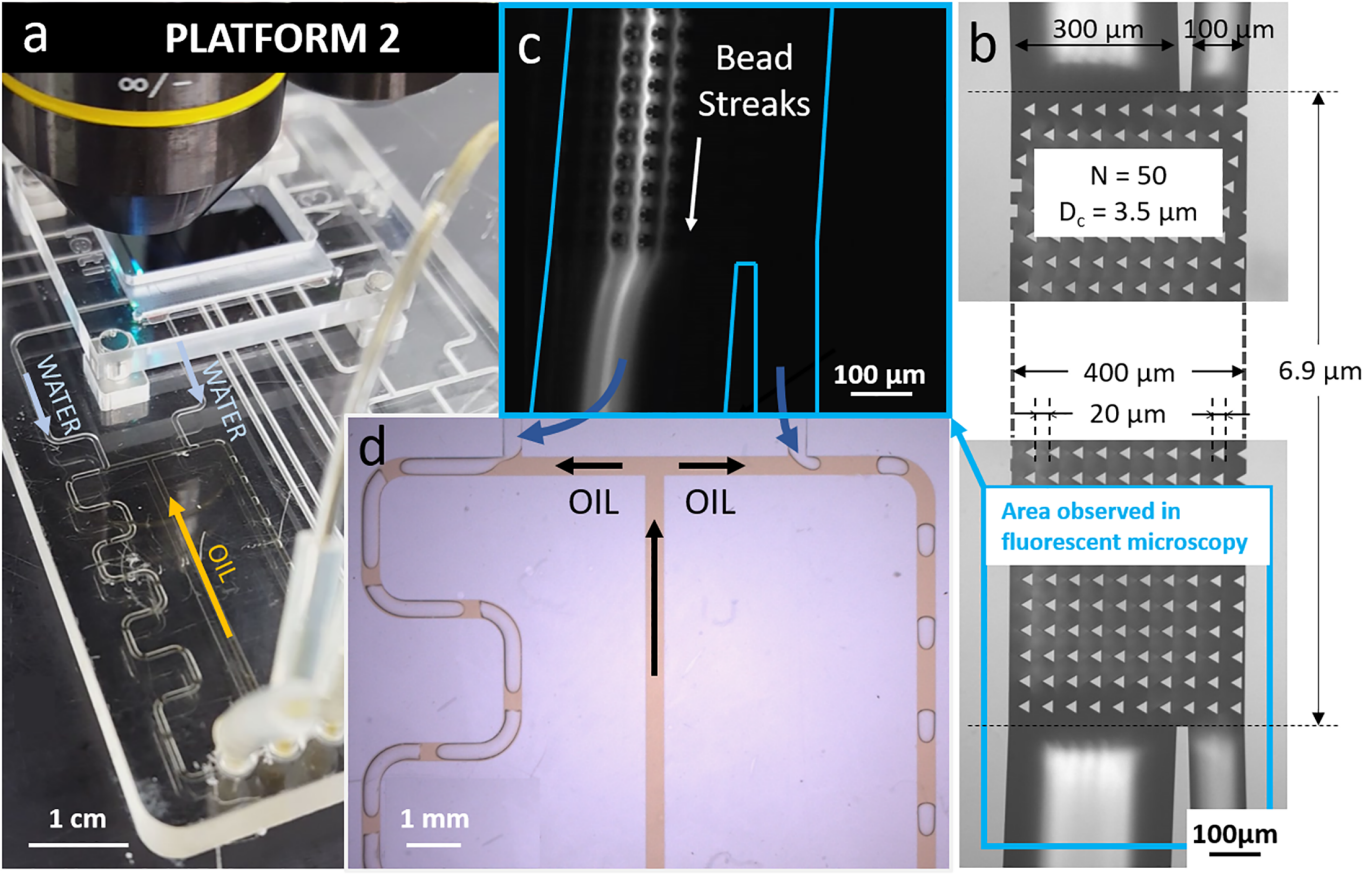
a) Photograph of platform 2 showing the DLD silicon chip connected to a plastic cartridge with two T-junctions supplied by the same oil inlet. b) Bright-field image of the DLD device with characteristic dimensions. The area located in the blue frame corresponds to the fluorescence image in c). c) Fluorescent streaks of 10 μm-beads at the DLD outlet. d) T-junctions connected to the DLD outlets.

In platform 2 (Fig 3), the T-junctions are dependent since the same oil inlet supplies both droplet generation areas. A longer serpentine channel is connected to the left DLD outlet, when compared to the right side, in order to mimic the connection of different hydraulic resistances to DLD outlets (Fig 3a). Thanks to the common oil inlet, both DLD backpressures are balanced at the same time, which is confirmed by the bead streak profile inside the DLD channel (Fig 3c).

Platform 2 is easier to control than platform 1 since only one oil inlet has to be controlled instead of two. However, it can be useful to control both oil inlets independently, especially when the connected microfluidic modules feature strongly different hydraulic resistances at the DLD outlets.

### Cascading DLD modules

**Fig 4:**
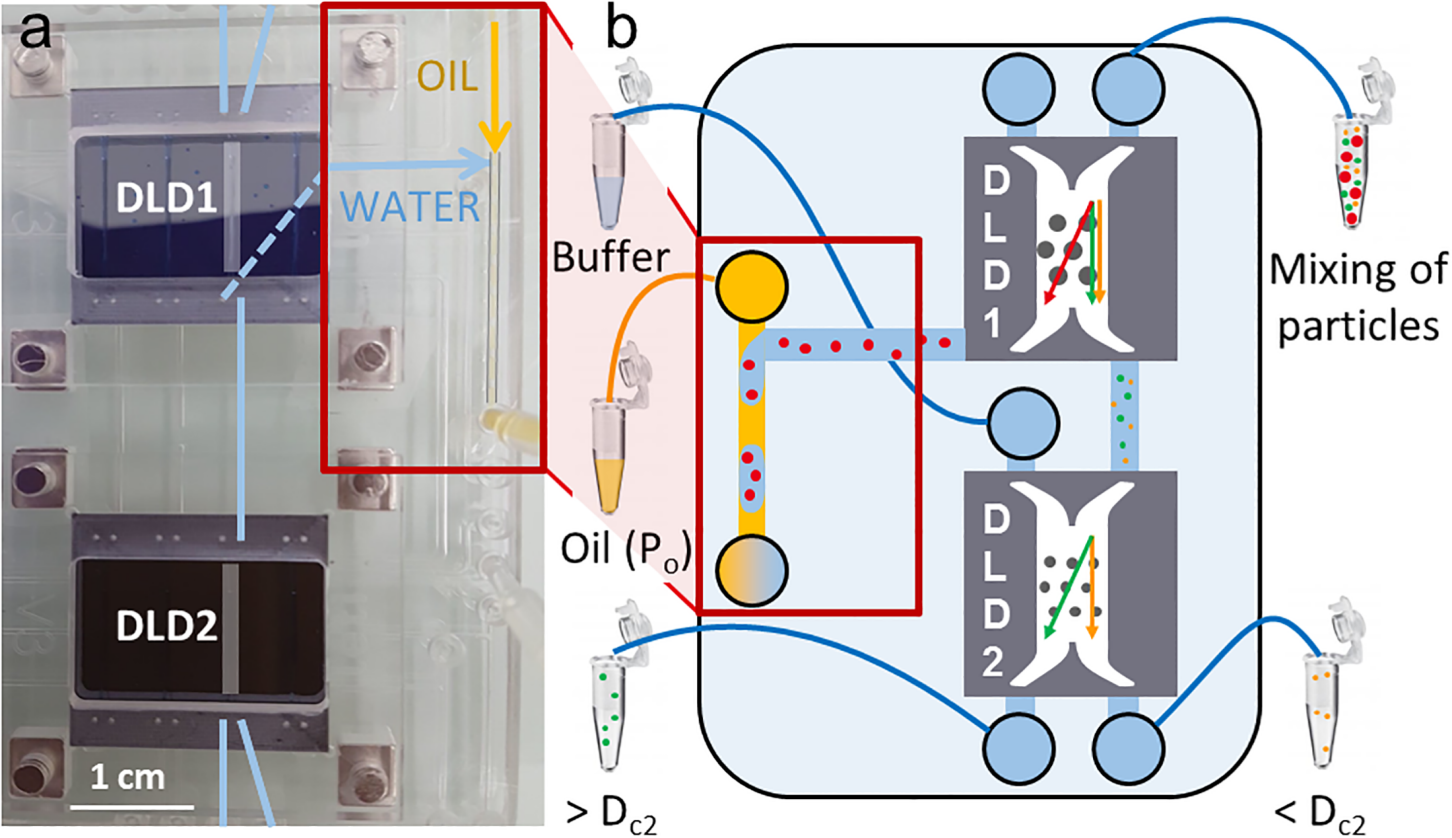
a) Photograph of platform 3, showing the connection of two DLD silicon chips (DLD1 and DLD2). One outlet of DLD1 is connected to the inlet of DLD2, while the other DLD1 outlet is connected to a T-junction in the cartridge. b) Schematic representation of platform 3. DLD1 deviates the largest red particles (> D_c1_), while DLD2 separates the green (> D_c2_) and the yellow (< D_c2_) particles. The red particles are encapsulated in a T-junction, supplied by an oil inlet at an input pressure P_o_.

A third platform is proposed to cascade DLD modules (Fig 4). A first DLD module called “DLD1” separates particles around a critical diameter D_c1_. DLD1 has two inlets—particle solution from the right inlet and buffer solution from the left inlet—and two outlets—non-deviated particles in the right outlet and deviated particles in the left outlet. Non-deviated particles from DLD1 (with diameters smaller than D_c1_) are injected into DLD2, for which the critical diameter D_c2_ is smaller than D_c1_. The other DLD1 outlet is connected to a T-junction that enables to balance the important hydraulic resistance presented by DLD2. In our experimental example, DLD1 has 60 μm triangular pillars (D_p_), 60 μm inter-pillar spacing (G) and an array periodicity (N) of 22, with a predicted critical diameter (D_c_) equal to 15 μm. DLD2 has the following parameters: D_p_ = 20 μm, G = 20 μm, N = 50 and D_c_ = 3.5 μm.

**Fig 5:**
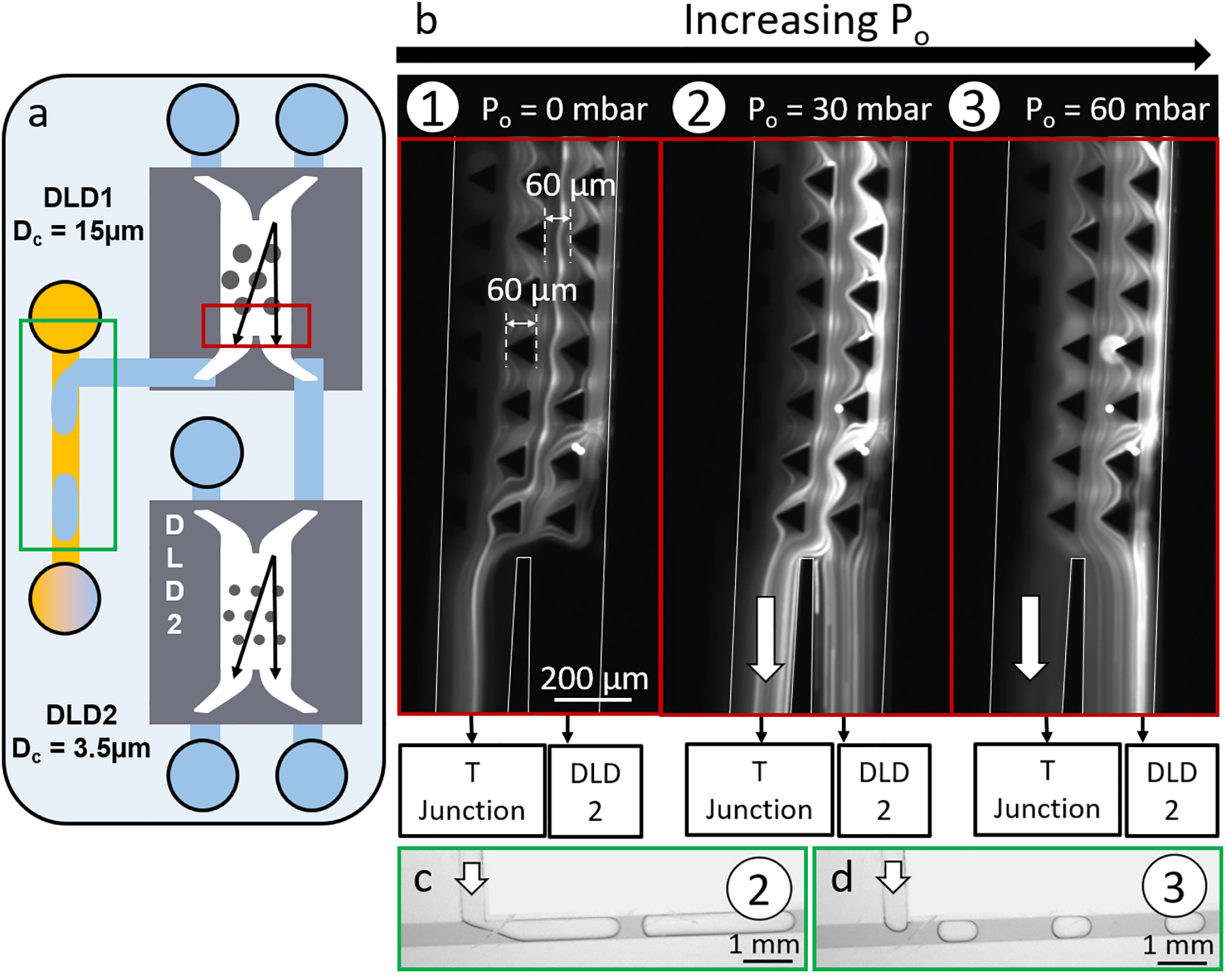
a) Schematic representation of the cascaded configuration: the area located in the red frame corresponds to the images in b) and the area located in the green frame corresponds to the images in c) and d). b) 10 μm-bead streaks at the DLD1 outlet for three values of the oil pressure at the T-junction (P_o_). The left outlet of DLD1 is connected to a T-junction, while the right outlet is connected to a DLD2 module. c) Droplet generation at the T-junction connected to the DLD1 left outlet in condition n°2 (P_o_ = 30 mbar). d) Droplet generation at the T-junction connected to the DLD1 left outlet in condition n°3 (P_o_ = 60 mbar).

Fig 5 illustrates how the input oil pressure at the T-junction (P_o_) influences the particle flow inside the DLD1 module, when DLD1 is connected to a DLD2 module. Fig 5b represents 10 μm-bead streaks at the DLD1 outlet. The right output channel is connected to DLD2, while the left output channel is connected to a T-junction. In the three presented conditions, the streaks are rather focused on the right side of the pillar channel because the 10 μm-beads are not deviated by the DLD1 module (D_c1_ = 15 μm, larger than the bead size).

When no pressure is applied on the oil solution at the T-junction (condition n°1, P_o_ = 0 mbar), all the streaks are deviated from the top right side to the bottom left side of the DLD channel, in order to flow towards the T-junction: in this case, DLD1 backpressures are not balanced. The beads should follow their trajectory towards the right outlet, but they are finally deviated towards the T-junction since this outlet presents a lower hydraulic resistance.

When the oil pressure is increased (condition n°2, P_o_ = 30 mbar), droplets start to be generated at the T-junction (Fig 5c) and some particles start to follow the correct trajectory towards DLD2.

By further increasing the oil pressure (condition n°3, P_o_ = 60 mbar), the droplet length decreases (Fig 5d), which means that the flow rate at the left DLD outlet decreases. At this P_o_ value, both hydraulic resistances are balanced at the DLD1 outlets, as shown by the bead streaks on Fig 5b.

These results illustrate the importance of choosing the correct oil pressure at the T-junction to efficiently handle the particle flow in the DLD modules. This specific oil pressure has to be adapted to the other input parameters of the platform, like the input pressures at the DLD modules and the hydraulic resistances of the chips and the cartridge. In order to anticipate the optimum pressures to apply to maximize the separation efficiency of the DLD platform, a predictive model has been established. This model takes into account all the input parameters of the platform and aims at determining what are the right pressure conditions to apply in any configuration, regardless of the DLD1 and DLD2 dimensions.

### Predictive model to optimize the platform control

**Fig 6:**
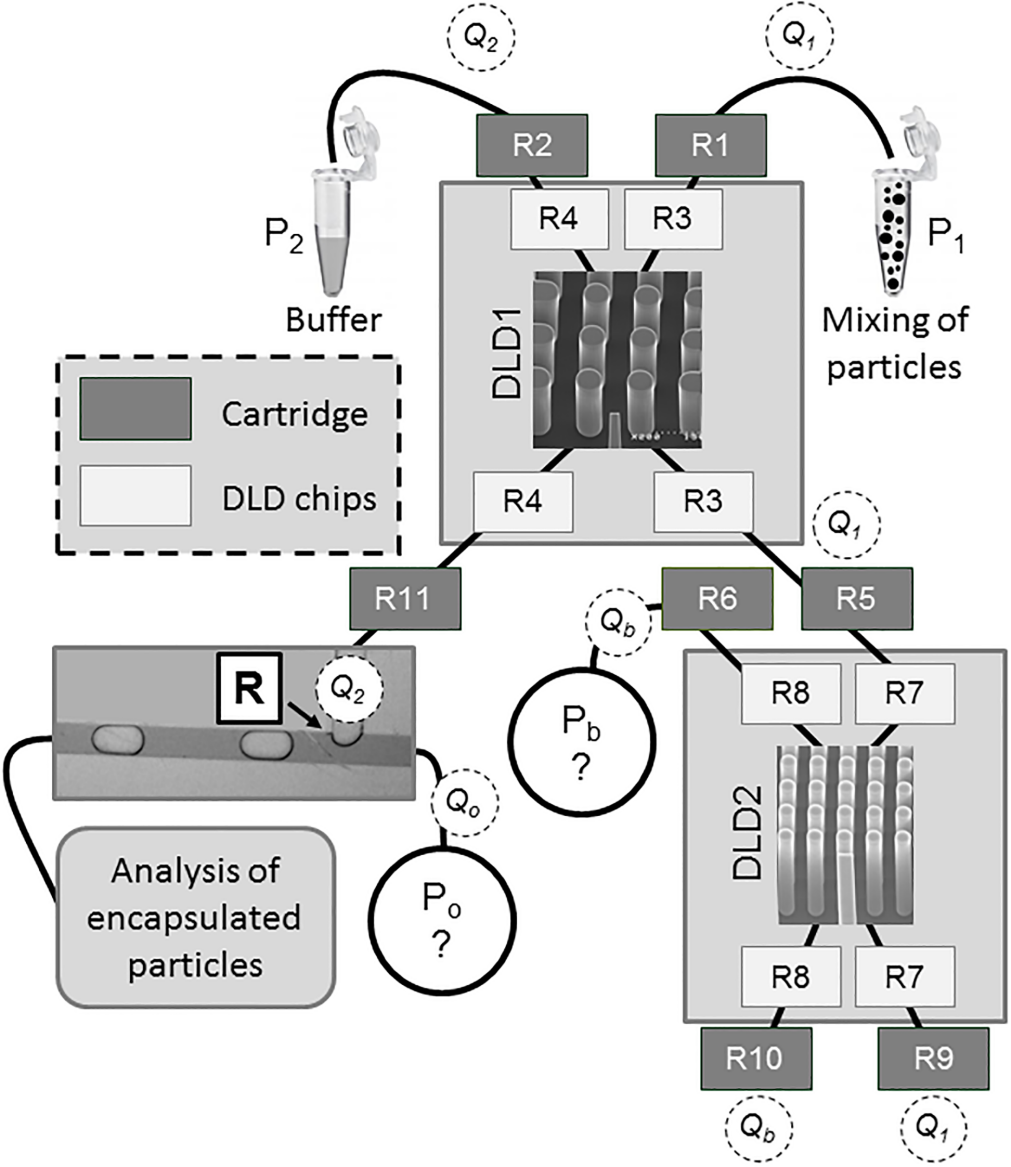
Modeling of platform 3: R1 to R11 represent the hydraulic resistances of the microchannels in the cartridge and in the DLD chips. P_1_ and P_2_ are the input pressures at the DLD1 entrance. P_b_ is the buffer pressure at the DLD2 entrance. P_o_ is the input oil pressure at the T-junction. Q_1_, Q_2_, Q_b_ and Q_o_ are the flow rates in the DLD channels and in the T-junction.

All the fluidic elements of platform 3 have been modeled by hydraulic resistances (Fig 6).

The input parameters of the model are the hydraulic resistances R1 to R11 and the pressures at the DLD1 entrance P_1_ and P_2_. The objective of the model is to give two optimum output parameters: the buffer pressure at the DLD2 entrance (P_b_) and the oil pressure at the T-junction (P_o_). Optimization of these output parameters is based on two conditions expressed in the DLD1 and DLD2 channels:

1. The average velocity amplitude is constant in the entire DLD1 channel width (local variations are observed because of the parabolic flow profile between pillars,[26] but the global velocity amplitude is the same from both DLD1 inlets):
  $$Q_{1}=v^{DLD1}S_{1}^{DLD1}$$ (1)
  $$Q_{2}=v^{DLD1}S_{2}^{DLD1}$$ (2)
  where Q_1_ and Q_2_ are the input flow rates at the DLD1 sample and buffer inlets respectively, v^DLD1^ is the flow velocity in the DLD1 channel, and S_1_^DLD1^ and S_2_^DLD1^ are the channel cross-sections at the DLD1 sample and buffer inlets respectively.
2. The same condition has to be applied at the DLD2 module:
  $$Q_{1}=v^{DLD2}S_{1}^{DLD2}$$ (3)
  $$Q_{b}=v^{DLD2}S_{2}^{DLD2}$$ (4)
  where Q_1_ and Q_b_ are the input flow rates at the DLD2 sample and buffer inlets respectively, v^DLD2^ is the flow velocity in the DLD2 channel, and S_1_^DLD2^ and S_2_^DLD2^ are the channel cross-sections at the DLD2 sample and buffer inlets respectively.

From the Hagen-Poiseuille law for a steady-state flow and an incompressible fluid, three additional relationships between the input pressures and the flow rates can be written:

$$P_{1}=(R_{1}+R_{3})Q_{1}+R_{DLD1}(Q_{1}+Q_{2})+(R_{3}+R_{5}+R_{7})Q_{1}+R_{DLD2}(Q_{1}+Q_{b})+(R_{7}+R_{9})Q_{1}$$ (5)

$$P_{2}=(R_{2}+R_{4})Q_{2}+R_{DLD1}(Q_{1}+Q_{2})+(R_{4}+R_{11}+R)Q_{2}$$ (6)

$$P_{b}=(R_{6}+R_{8})Q_{b}+R_{DLD2}(Q_{1}+Q_{b})+(R_{8}+R_{10})Q_{b}$$ (7)

The key approach here is to consider droplet generation at the T-junction as contributing as an additional constant hydraulic resistance R that is applied on the DLD1 left outlet.

**Fig 7:**
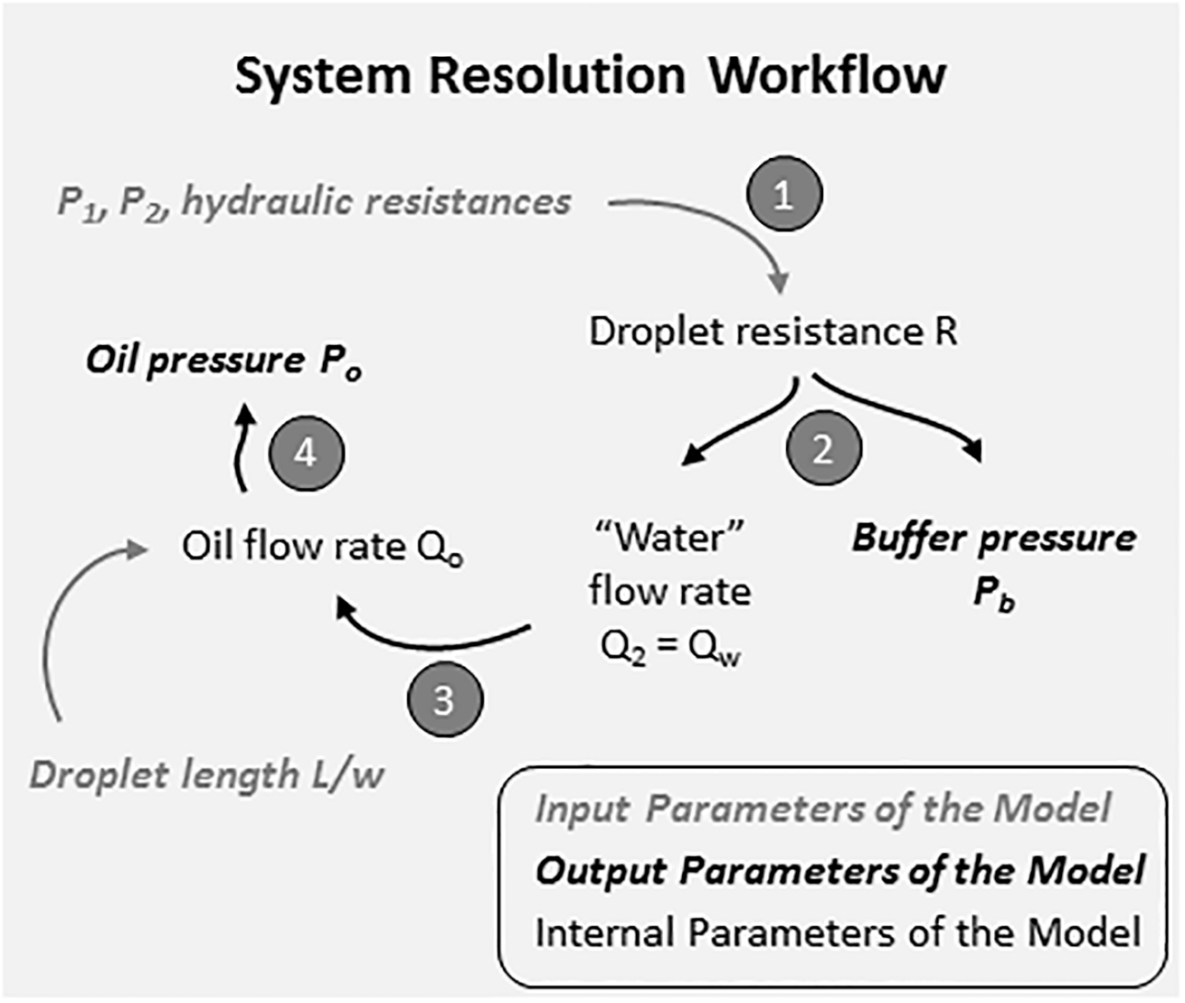
Schematic of the successive resolution steps.

By using the two conditions and Eqs (5), (6) and (7), the unknown input pressures (P_b_ and P_o_) can be determined with the system resolution workflow described on Fig 7.

**Fig 8:**
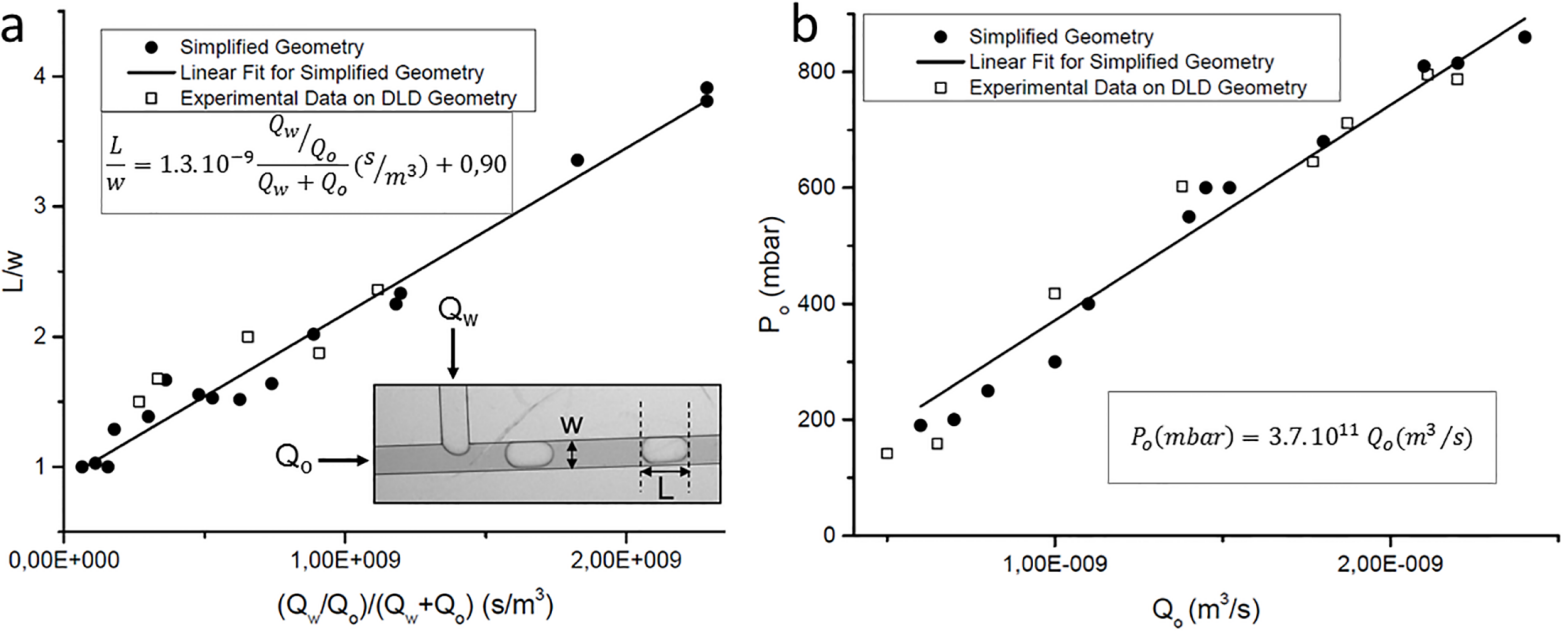
a) Experimental relation between the length ratio of the droplets (L/w, where w is the channel width) and the flow rate ratio (where Q_w_ and Q_o_ are the water and oil flow rates respectively). A linear fit is proposed from data obtained with a simplified T-junction geometry without DLD modules. This relation is then verified with the complete DLD cascaded platform, including the same T-junction geometry. b) Experimental relation between the oil input pressure P_o_ and the oil flow rate Q_o_ at the T-junction. The linear fit is again obtained from the simplified T-junction geometry and verified with the complete platform.

Step 1 The droplet resistance R at the T-junction is determined by solving the following equation, obtained from the two conditions and the Eqs (5), (6) and (7) presented above:
  P1(1−11+γ(R)βη)+P2(γ(R)Xξ(R)+RDLD1+α(R)1+γ(R)ηβ−α(R))=0 (8)
  where X=S2DLD1S1DLD1 and x=S2DLD2S1DLD2
  $$\alpha (R)=\frac{R_{DLD1}}{R_{2}+2R_{4}+R_{11}+R_{DLD1}+R}$$
  $$\gamma (R)=R_{1}+2R_{3}+R_{5}+2R_{7}+R_{9}+R_{DLD1}+R_{DLD2}-\frac{R_{DLD1}^{2}}{R_{2}+2R_{4}+R_{11}+R_{DLD1}+R}-\frac{R_{DLD2}^{2}}{R_{6}+2R_{8}+R_{10}+R_{DLD2}}$$
  $$\xi (R)=R_{2}+2R_{4}+R_{11}+R_{DLD1}+R$$
  $$\beta =\frac{R_{DLD2}}{R_{6}+2R_{8}+R_{10}+R_{DLD2}}$$
  $$\eta =x(R_{6}+2R_{8}+R_{10}+R_{DLD2})+R_{DLD2}$$
Step 2 After determining the droplet resistance R, the buffer pressure P_b_ and the water flow rate at the T-junction Q_2_ can be determined as follows:
  Pb=P1−α(R)P2β+γ(R)η (9)
  Q2=P2RDLD1X+ξ(R) (10)
Step 3 The oil flow rate Q_o_ is then determined from the water flow rate Q_2_ (denoted Q_w_ in the following sections). The relationship between Q_o_ and Q_w_ was first determined from a simplified T-junction geometry that has the same geometry as the T-junction of platform 3, but without DLD modules. The oil flow rate Q_o_ was experimentally determined (by measuring the weight loss of the oil tube during injection) to obtain the fitting data in the simplified geometry. A linear relationship was found between the droplet length ratio Lw and the flow rate ratio QwQoQw+Qo (Fig 8a), with the slope 1.3.10^−9^ ± 0.2.10^−9^ (m^3^/s) and the intercept 0.9 ± 0.1. This linear trend is in agreement with the model of Zhang et al.[43] Our model was obtained with the same oil and water solutions as for DLD experiments in order to get the correct fitting pre-factor. However, Garstecki et al. [42] showed that this factor is independent of the viscosity and interfacial tension of the fluids for low capillary numbers, which is the case in the squeezing regime that is used in our platform. The relationship found in the simplified geometry was verified in the entire platform of Fig 4, by measuring the corresponding droplet length when changing the water and oil flow rates at the T-junction. Therefore, when using this platform, the oil flow rate to apply is determined from the calculated water flow rate (Q_2_ from step 2 of the resolution workflow in Fig 7) and from the chosen droplet length. Thus, an additional advantage of this platform is the ability to tune the droplet size that can be adapted to the requirements of the connected downstream module.
Step 4 If the system is controlled with input pressures instead of flow rates, an additional relationship is required to find the oil pressure P_o_ from the oil flow rate Q_o_. This relation was first determined with the simplified T-junction geometry, by measuring the obtained flow rate Q_o_ when applying different pressure values P_o_. The obtained linear fitting parameter (3.7.10^11^ ± 0.6.10^11^ mbar.s/m^3^) was then verified with the complete DLD platform (Fig 8b).

### Validation of the model on the complete platform

**Fig 9:**
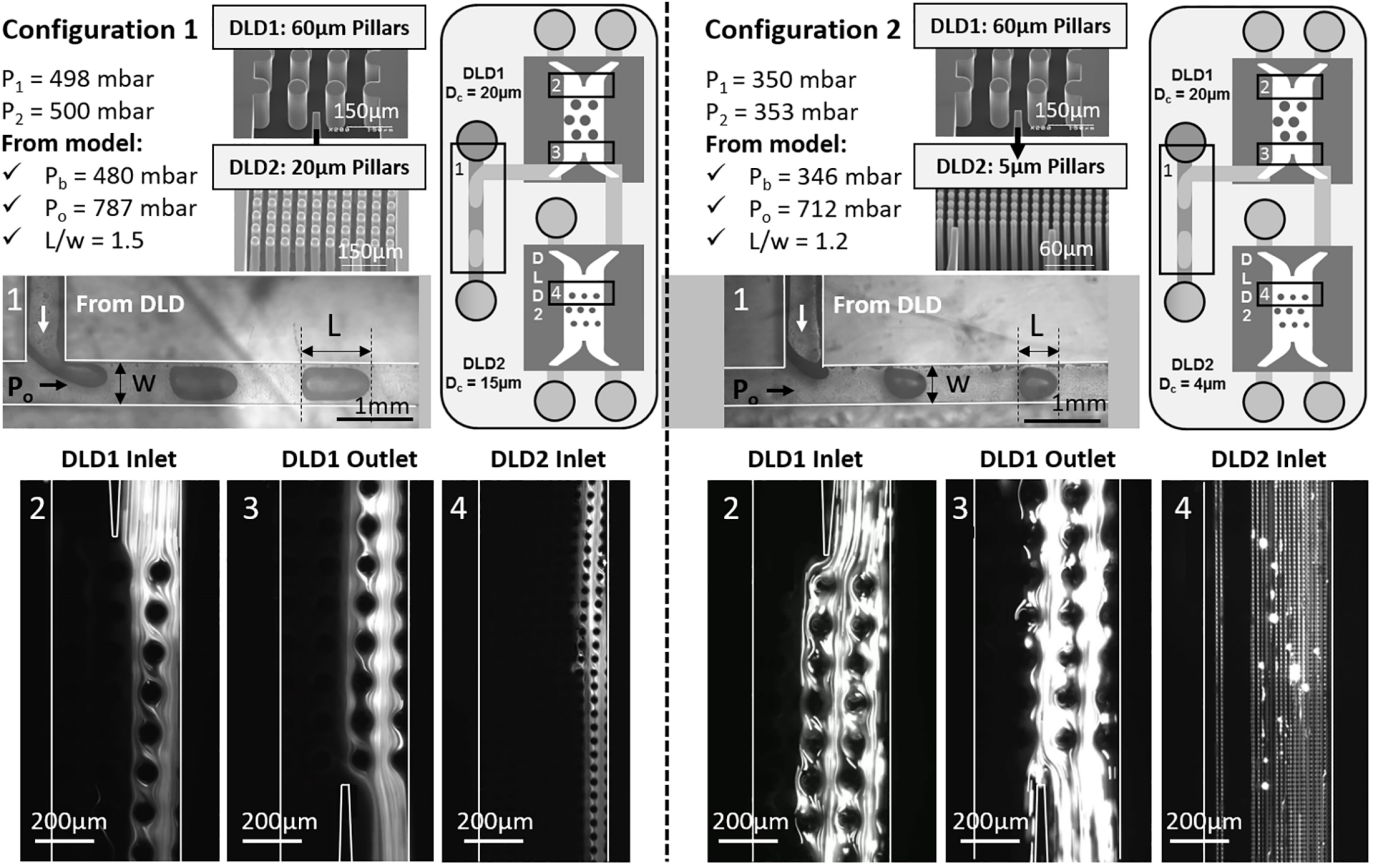
Validation of the model on the complete DLD platform in two configurations. 10 Configuration 1 has a DLD1 module with 60 μm pillars (D_c1_ = 20 μm) and a DLD2 module with 20 μm pillars (D_c2_ = 15 μm), while configuration 2 has the same DLD1 module (D_c1_ = 20 μm) but a DLD2 module of 5 μm pillars (D_c2_ = 4 μm). 10 μm-beads are injected in configuration 1, while 5 μm-beads are injected in configuration 2. Input pressures P_1_ and P_2_ at the DLD1 entrance are chosen; then the buffer pressure P_b_ and oil pressure P_o_ are calculated from the proposed model, by choosing a given droplet size ratio L/w. In both cases, the validity of the model is verified by measuring the droplet length at the T-junction (area n°1) and the particle distribution at the DLD1 inlet (area n°2) and outlet (area n°3) and at the DLD2 inlet (area n°4). Areas 1 to 4 are located on the schematic representations of the cascaded platform for both configurations.

The proposed model was used to determine the optimum input parameters of the complete DLD platform in several configurations. In the first configuration (configuration 1 in Fig 9), the DLD1 module features a pillar array of 60 μm circular pillars with 60 μm inter-pillar spacings and array periodicity of 22 (critical diameter of 20 μm) and the DLD2 module has 20 μm circular pillars with 20 μm inter-pillar spacings and array periodicity of 6 (critical diameter of 15 μm). The second configuration (configuration 2 in Fig 9) has the same DLD1 module but a different DLD2 module with 5 μm circular pillars, 5 μm inter-pillar spacings and an array periodicity of 8 (critical diameter of 4 μm).

All the individual DLD devices have been characterized elsewhere [27]. After choosing the input pressures at the DLD1 entrance (P_1_ and P_2_), our model resolution workflow (Fig 7) was implemented to determine the other input parameters of the platform: the buffer pressure at the DLD2 entrance (P_b_) and the oil pressure at the T-junction (P_o_), for a chosen droplet size (Lw). The obtained values for P_b_ and P_o_ are given in Fig 9 for both configurations. Then these pressure values were applied on the platform in each configuration to verify the agreement between our predictive model and experimental droplet size and particle trajectory (Fig 9). The stability of droplet generation was verified in both configurations with the predicted pressure conditions, as droplets were formed in the squeezing regime. However, large flow rate ratio QwQo should be avoided to maintain the droplet breakup process at the channel intersection and avoid droplet generation instabilities and parallel flows [44]. 10 μm-fluorescent beads were injected in the platform in configuration 1, while 5 μm-beads were used in configuration 2. Therefore, according to the critical diameter of each module, beads are expected to follow a zigzag trajectory in both DLD modules for configuration 1, and they are expected to be displaced only by the second DLD module in configuration 2. So, in both configurations, when the system is well balanced, beads should flow out of the DLD1 module from the same side as at the entrance. This was verified when the predicted input pressures were applied on the platform (Fig 9): beads have a zigzag trajectory along the DLD1 module and flow out from DLD1 through the correct outlet on the DLD1 right side, before entering into the DLD2 module. In the DLD2 stage, it is verified that the particle trajectory is in accordance with predictions (zigzag mode in configuration 1 and displacement mode in configuration 2), which means that the DLD2 input buffer pressure is well adapted to the bead flow rate at the DLD2 entrance. Thus, the proposed model efficiently anticipates the required input pressures to optimize the flow distribution in both DLD modules of the cascaded platform.

## Conclusion

Droplet generation in T-junctions was proposed as a solution to balance backpressures of DLD modules. Pressure balancing at DLD outlets is essential to ensure high separation efficiency. While usual balancing methods require either a specific chip design or a waste syringe pump, here we propose a completely modular and reconfigurable platform to cascade DLD modules together or to other analysis steps. By connecting T-junctions to DLD outlets, droplet generation ensures at the same time pressure balancing and encapsulation of the sorted particles.

In order to anticipate the optimum pressures to apply on the different modules of the platform, a general model was proposed for a configuration of two cascaded DLD steps. Thanks to this model, the separation efficiency of the cascaded platform was optimized with any DLD modules. This capacity enables a modular approach, where the same platform and its predictive model can be used for a wide range of applications, with different DLD dimensions.

Moreover, pressure balancing through droplet generation can be generalized to any multi-step microfluidic devices. Indeed, droplet formation enables to connect two—or more, providing the connection to corresponding T-junctions—successive microfluidic steps without interference between each other. This is particularly useful when the first step needs a constant output pressure at all the different outlets, and the second step requires particle encapsulation. Based on the same approach as presented in this paper, new models could be implemented to anticipate the optimum balancing pressure to apply on the continuous phase of the T-junction.
